# The impact of social participation on the quality of life among older adults in China: a chain mediation analysis of loneliness, depression, and anxiety

**DOI:** 10.3389/fpubh.2024.1473657

**Published:** 2024-09-25

**Authors:** Lu-Yin Liang

**Affiliations:** Law School, Guangdong University of Technology, Guangzhou, China

**Keywords:** social participation, loneliness, depression, anxiety, quality of life, older adult, China

## Abstract

This cross-sectional study investigates the impact of social participation on the quality of life (QOL) among older adults in China. Using convenience sampling, data were collected from 508 individuals aged 60 and above (M_age = 70.53 ± 7.90 years; 56.5% women). Statistical analyses were conducted using SPSSAU software, including Pearson correlation analysis to assess relationships between social participation, psychological health indicators (loneliness, depression, and anxiety), and QOL. Multiple regression analysis and chain mediation analysis were subsequently performed to explore the mediating effects of loneliness, depression, and anxiety on the relationship between social participation and QOL. The results indicated significant correlations between social participation and loneliness (*r* = −0.313, *p* < 0.001), depression (*r* = −0.487, *p* < 0.001), anxiety (*r* = −0.305, *p* < 0.001), and QOL (*r* = 0.476, *p* < 0.001). The mediation analysis revealed significant chain mediation effects of loneliness, depression, and anxiety on the relationship between social participation and QOL (*β* = 0.006, *p* < 0.001, 95% CI [0.001, 0.007]). Higher levels of social participation were associated with lower levels of loneliness, which in turn reduced depression and anxiety, thereby enhancing QOL. These findings highlight the importance of promoting social participation to improve psychological wellbeing and QOL among older adults in China. The study advocates for active social engagement and the provision of relevant services, as well as psychological support and emotional counseling for those facing mental health challenges due to insufficient social participation.

## 1 Introduction

With continuous socioeconomic development and advancements in living standards and healthcare, life expectancy is consistently increasing. According to the “World Social Report 2023” published by the United Nations, the global population aged 65 and above reached 761 million in 2021. This number is expected to increase to 1.6 billion by 2050 (1). Among all nations, China stands out with the largest population of older adults, experiencing a rapid acceleration in population aging processes related to later life. Data from the National Bureau of Statistics of China reveals that by the end of 2023, the population aged 60 and above had reached 296.97 million, representing 21.1% of the total population (2).

China has placed significant emphasis on the health of older adults in its efforts to embrace the opportunities presented by a growing aging population. Drawing on the governance experiences of the international community, China has timely proposed the implementation of a national strategy for active aging. The WHO defines Active Aging as “the process of optimizing opportunities for health, participation, and security in order to enhance the quality of life as people grow older” (3). This concept clearly elucidates that active engagement and the maintenance of healthy functioning are key objectives in promoting the wellbeing of older adults.

Unlike the traditional Chinese view of care for older adults, which focuses primarily on “Lao You Suo Yang” (having care in old age) and “Lao You Suo Yi” (being dependent in old age), active aging further emphasizes “Lao You Suo Yong” (being useful in old age) and “Lao You Suo Wei” (being active in old age) (4). It advocates for a positive attitude toward later life from both the public and the older adults themselves. In this context, social participation by older adults is a key component of active aging and an important means of demonstrating the value of the older population (5). Social participation is defined as an individual’s involvement in activities that facilitate interaction with others in society or the community, expressing interpersonal interactions outside the home (6–9). This includes active engagement in small social circles, such as friendships, as well as larger societal contexts, like workplace interactions (10), and the satisfaction derived from these engagements (11).

From both a theoretical standpoint and the United Nations’ development strategy for older adults, there is clear and strong support for the importance of social participation among older adults. According to the activity theory, one of the three major historically significant theories of psychosocial development in old age and successful aging, active aging results from older adults staying active, particularly in social participation, and engaged within society (12–14). This theory suggests that older adults should assume new roles to replace those lost due to retirement. Engaging in new activities helps mitigate the negative emotions associated with the disruption of social roles, thereby reducing their distance from society (15). By maintaining personal relationships and staying socially active, older adults can slow down or even avoid age-related losses and improve their subjective wellbeing, particularly life satisfaction. To sustain this wellbeing, older adults should substitute previous life roles, relationships, and activities from their mid-life phase (e.g., work, caring for children) with new ones (e.g., volunteering, grand-parenting) (16). This approach aligns with the United Nations’ “Active Ageing” strategy, which emphasizes that social participation is a legitimate right of the older adults and should be supported and safeguarded (17).

Existing studies have empirically analyzed the impact of social participation on various aspects of psychological health in older adults. Regarding the influence of social participation on loneliness in older adults, a cross-sectional study involving 29,795 older individuals found that those who actively engaged in social participation were less likely to experience feelings of loneliness (18). Similarly, longitudinal research has confirmed a significant negative correlation between social participation and reduced loneliness in older adults (19–21). Furthermore, intervention studies have indicated that interventions aimed at increasing social participation can effectively enhance the social support received by older adults, thereby significantly reducing their feelings of loneliness (22–24). The relationship between social participation and symptoms of anxiety and depression has been extensively discussed in gerontological research. Both cross-sectional and longitudinal studies generally support the notion that lower levels of social participation in older adults are associated with more severe symptoms of anxiety and depression (25–28). In the contemporary era of rapid information technology development, social participation can be conducted not only through traditional offline interactions but also via online communication, yielding positive health outcomes. For instance, a study by Hofer and Hargittai (29) found that online social participation can also help alleviate anxiety and depression among older adults. Some scholars argue that the relationship between social participation and emotional distress is likely bidirectional (30). That is, increased emotional distress can exacerbate symptoms of anxiety and depression, such as irritability, social avoidance, withdrawal, anhedonia, and negative cognitive biases, leading to reduced or unsatisfactory social interactions (31). Conversely, heightened sensitivity to social rejection resulting from poor social participation may also intensify symptoms of anxiety and depression.

Despite having the world’s largest older population, China has a dearth of research investigating the relationship between social participation, psychological health, and life satisfaction among older adults. Only a limited number of studies have explored the connections between social participation and feelings of loneliness (19, 32), depression (33, 34), anxiety (35), and both QOL and life satisfaction (36, 37). These investigations, however, tend to focus on single health indicators and fail to provide a comprehensive analysis of how psychological health may act as an internal mechanism influencing the relationship between social participation and life satisfaction. Notably, a study of older adults in Chinese nursing homes has demonstrated that depression mediates the relationship between loneliness and QOL, indicating that loneliness can impact QOL through the mediating effect of depression (38). Additionally, a longitudinal study in Hong Kong found that social participation mediates the relationship between loneliness and QOL among older adults (32). However, it remains unclear whether social participation can influence QOL through loneliness, depression, and anxiety individually, and whether loneliness can further mediate the effect on depression, which in turn affects anxiety levels, ultimately influencing QOL. These gaps in the literature highlight the need for further research.

To enrich the empirical knowledge on social participation among older adults in China and address current research gaps, this study aims to provide insights into the underlying mechanisms elucidating the relationship between social participation and QOL among older Chinese. This research first analyzes the relationship between social participation and QOL among older adults in China. Subsequently, it examines the mediating roles of loneliness, depression, and anxiety in the relationship between social participation and QOL. Furthermore, it assesses whether social participation influences QOL through a chained mediation process involving loneliness, depression, and anxiety. Based on these theoretical considerations and research objectives, the following hypotheses are proposed:

*Hypothesis 1*: Social participation can positively predict the QOL among older adults in China.

*Hypothesis 2*: Social participation can indirectly predict the QOL among older adults in China through the intermediary role of loneliness.

*Hypothesis 3*: Social participation can indirectly predict the QOL among older adults in China through the intermediary role of depression.

*Hypothesis 4*: Social participation can indirectly predict the QOL among older adults in China through the intermediary role of anxiety.

*Hypothesis 5*: Social participation can indirectly predict the QOL among older adults in China through the chain mediating effect of loneliness, depression and anxiety.

## 2 Methods

### 2.1 Participants and procedure

This study involved 508 older adults aged 60 and above, drawn from 13 community service centers located across four administrative districts in Guangzhou, China: Yuexiu (three centers), Huadu (two centers), Nansha (five centers), and Panyu (three centers). These service centers are funded by the local government and provide various routine day services for older adults in the community. The study employed a convenience sampling method. With institutional consent, staff at the centers invited older adults who were either participating in community activities or receiving in-home support services to complete a questionnaire. The inclusion criteria were: participants had to be 60 years or older, mentally alert, capable of independently completing the questionnaire, and willing to provide informed consent after being briefed on the study’s purpose and procedures. Exclusion criteria included being under 60 years of age, having severe cognitive impairments or serious mental illnesses, and experiencing difficulties in understanding or communicating effectively during the questionnaire process.

The study utilized two data collection methods to accommodate the preferences and circumstances of the older adult participants. Some older adults completed the paper-based survey with the assistance of staff at the service centers, who facilitated the in-person administration of the survey. Additionally, an electronic version of the survey was made available, designed and distributed using “Questionnaire Star,” an online crowdsourcing platform in China comparable to Amazon Mechanical Turk. This dual approach ensured a comprehensive and accessible means of gathering data from participants. Participants were fully informed of the research purpose, data confidentiality process, and their rights and obligations before data collection, with the survey taking approximately 20 min to complete. Informed consent was obtained from all participants, who signed a consent sheet if they agreed to participate.

The study was approved by the Human Research Ethics Committee of Guangdong University of Technology. Out of 521 questionnaires collected, 13 were excluded due to invalid responses or non-qualifying ages, resulting in 508 valid questionnaires and a response rate of 97.5%.

### 2.2 Measures

This study employed four scales to measure the variables of interest: the social participation items from the China General Social Survey (CGSS), the Short-Form Measure of Loneliness (ULS-8), the Hospital Anxiety and Depression Scale (HADS), and the Quality of Life Scale (QOLS). In addition, demographic information was collected from respondents, including age, sex, living arrangement, educational attainment, marital status, and economic status. In the chain mediation model analyses, age, sex, living arrangement, educational attainment, marital status, economic status were controlled to account for potential confounding biases.

#### 2.2.1 Social participation

Social participation was measured using relevant items from the China General Social Survey (CGSS), a comprehensive and household-based continuous social survey in China. The questionnaire assessed social participation among older adults in three main areas: participation in entertaining activities, gatherings with relatives and friends, and involvement in cultural activities. The questionnaire included 12 items rated on a five-point scale: “Never,” “Several times a year or less,” “Several times a month,” “Several times a week,” and “Daily,” assigned values of 1, 2, 3, 4, and 5, respectively. Higher scores indicated greater levels of social participation among older adults (39, 40). The internal reliability of the scale in the current sample was acceptable, with a Cronbach’s alpha coefficient of 0.77.

#### 2.2.2 Loneliness

The loneliness of older Chinese participants was evaluated using the Short-Form Measure of Loneliness (ULS-8) (41). This scale consists of eight items, each rated on a four-point Likert scale ranging from “never” to “always.” The total score is calculated by summing the responses to all items, with higher scores reflecting a greater degree of loneliness. In the current sample, the internal reliability of the ULS-8 was found to be good, with a Cronbach’s alpha coefficient of 0.87.

#### 2.2.3 Depression and anxiety

The Hospital Anxiety and Depression Scale (HADS) (42) was utilized to assess symptoms of anxiety and depression among older Chinese participants. This self-reporting tool consists of 14 items, with seven items dedicated to measuring anxiety severity (HADS-A) and another seven items measuring depression severity (HADS-D). Each item is scored on a scale from 0 to 3, yielding a possible total score range of 0–21 for both HADS-A and HADS-D. Higher scores indicate more pronounced symptoms of anxiety or depression. The internal consistency of the scale in this study was acceptable, with a coefficient alpha of 0.80 (0.73 for HADS-A and 0.66 for HADS-D).

#### 2.2.4 QOL

The Quality of Life Scale (QOLS) developed by Qiu (43) was used to measure the QOL among older Chinese participants. This scale encompasses 21 items across four dimensions: physical health, psychological wellbeing, social support, and environmental factors. Each item is rated on a five-point Likert scale with response options ranging from “strongly agree” to “strongly disagree,” scored as 5, 4, 3, 2, and 1, respectively. Higher scores indicate a greater level of agreement with the statements, reflecting a higher QOL. In the current sample, the Cronbach’s alpha coefficient for the scale was 0.960, indicating excellent reliability.

### 2.3 Data analyses

This study primarily utilized SPSSAU software for data processing and analysis. After excluding missing values and data not meeting the inclusion criteria (participants under 60 years of age), a descriptive analysis was conducted. The descriptive statistics covered age, sex, living arrangement, educational attainment, marital status, and economic status of the surveyed older adults.

Subsequently, Pearson correlation analysis and multiple regression analysis were performed to examine the relationships between the major variables. Upon identifying significant associations between the measured variables, a chain mediation analysis was conducted to investigate the mediating effects of loneliness, depression, and anxiety on the relationship between social participation and QOL. To estimate the mediation effects, 1,000 bootstrap samples were extracted. The significance of the regression coefficients was tested using bootstrap 95% confidence intervals. If the 95% confidence interval did not include zero, the corresponding effect was considered significant, indicating a valid mediation effect.

## 3 Results

A total of 508 older Chinese respondents participated in this survey, with ages ranging from 60 to 98 years (M = 70.53, SD = 7.90). The sample consisted of 221 males (43.5%) and 287 females (56.5%). The majority of respondents lived with family members (74.41%), while 25.59% lived alone. Regarding educational attainment, 59.06% had a junior high school education or below, and 40.94% had a senior high school education or above. Marital status was distributed as follows: 65.94% were married, 22.64% were widowed, 5.12% were single, and 5.71% were divorced. In terms of economic status, 21.85% of respondents had a monthly pension below 2,000 RMB, 41.73% had a pension between 2,000 and 5,000 RMB, and 36.42% had a pension exceeding 5,000 RMB (Table 1).

**Table 1:** Descriptive statistics.

Demographic variable	Category	No. of participants	%
Sex	Male	221	43.50
	Female	287	56.50
Living arrangement	Living alone	130	25.59
	Not living alone	378	74.41
Educational attainment	Never attend school	58	11.42
	Elementary school	122	24.02
	Junior high school	120	23.62
	Senior high school and above	208	40.94
Marital status	Married	335	65.94
	Singled	26	5.12
	Widowed	115	22.64
	Divorced	29	5.71
	Other	3	0.59
Economic status	<2000 RMB	111	21.85
	2000–5,000 RMB	212	41.73
	>5,000 RMB	185	36.42
Total		508	100.0

Table 2 displays the mean, standard deviation, and correlation coefficients of social participation, loneliness, depression, anxiety, and QOL. The results suggest that all the measured variables are significantly correlated with each other. Social participation was negatively related to loneliness (*r* = −0.313, *p* < 0.001), depression (*r* = −0.487, *p* < 0.001) and anxiety (*r* = −0.305, *p* < 0.001), and positively related to QOL (*r* = 0.476, *p* < 0.001). Loneliness was positively related to depression (*r* = 0.628, *p* < 0.001) and anxiety (*r* = 0.612, *p* < 0.001), and negatively related to QOL (*r* = −0.642, *p* < 0.001). Depression was positively related to anxiety (*r* = 0.561, *p* < 0.001), and negatively related to QOL (*r* = −0.652, *p* < 0.001). Anxiety was negatively related to QOL (*r* = −0.560, *p* < 0.001).

**Table 2:** Means, standard deviations and Pearson’s correlations of all variables.

	Mean	SD	1	2	3	4	5
Social participation	30.730	7.692	1				
Loneliness	16.844	5.157	−0.313***	1			
Depression	8.020	3.508	−0.487***	0.628***	1		
Anxiety	8.978	3.781	−0.305***	0.612***	0.561***	1	
QOL	75.480	15.108	0.476***	−0.642***	−0.652***	−0.560***	1

***p < 0.001.

Table 3 presents the results from the mediation analysis examining the roles of loneliness, depression, and anxiety in the relationship between social participation and QOL, controlling for age, sex, living arrangement, educational attainment, marital status, and economic status. In the first step, the direct effect of social participation on QOL was significant (*β* = 0.702, *p* < 0.001). Educational attainment, marital status, and economic status emerged as significant control variables. These findings support Hypothesis 1. In the second step, social participation was found to significantly predict loneliness (*β* = −0.100, *p* < 0.001). Educational attainment and marital status also displayed significant effects in this model. In the third step, both social participation (*β* = −0.130, *p* < 0.001) and loneliness (*β* = −0.366, *p* < 0.001) were significant predictors of depression, with age being the only significant control variable. In the fourth step, anxiety was significantly predicted by loneliness (*β* = 0.287, *p* < 0.001) and depression (*β* = 0.255, *p* < 0.001), but not by social participation (*β* = −0.011, *p* > 0.05). Sex was the sole significant control variable. Finally, when social participation (*β* = 0.384, *p* < 0.001), loneliness (*β* = −0.888, *p* < 0.001), depression (*β* = −1.047, *p* < 0.001), and anxiety (*β* = −0.670, *p* < 0.001) were included in the analysis, all these variables were found to have significant effects on QOL.

**Table 3:** Results of mediation analysis (*N* = 508).

Independent variables	Fitting index			Significance			95%CI	
	*R^2^*	*Adjust R ^2^*	F	*β*	SE	t	LLCI	ULCI
Dependent variable: QOL (total effect)								
Constant	0.297	0.288	35.244***	56.701	8.154	6.954**	0.538	0.866
Age				−0.154	0.082	−1.880		
Sex				1.847	1.173	1.575		
Living arrangement				2.865	1.636	1.751		
Educational attainment				1.268	0.581	2.184*		
Marital status				−2.087	0.687	−3.038**		
Economic status				7.042	2.354	2.991**		
Social participation				0.702	0.084	8.382**		
Dependent variable: loneliness								
Constant	0.233	0.223	25.318***	24.456	2.907	8.412**	18.744	30.168
Age				0.019	0.029	0.635	−0.039	0.076
Sex				−0.772	0.418	−1.845	−1.593	0.050
Living arrangement				−2.001	0.583	−3.430***	−3.148	−0.855
Educational attainment				−0.880	0.207	−4.252***	−1.287	−0.474
Marital status				0.847	0.245	3.460***	0.366	1.328
Economic status				−0.152	0.339	−0.448	−0.817	0.513
Social participation				−0.100	0.030	−3.348***	−0.159	−0.041
Dependent variable: depression								
Constant	0.514	0.507	75.502***	1.761	1.814	0.971	−1.804	5.326
Age				0.074	0.017	4.350***	0.041	0.108
Sex				−0.276	0.245	−1.125	−0.757	0.206
Living arrangement				0.402	0.345	1.164	−0.276	1.079
Educational attainment				−0.193	0.123	−1.571	−0.435	0.049
Marital status				0.050	0.145	0.348	−0.234	0.335
Economic status				0.058	0.198	0.294	−0.331	0.447
Loneliness				0.366	0.026	14.040***	0.315	0.418
Social participation				−0.130	0.176	−7.346***	−0.164	−0.095
Dependent variable: anxiety								
Constant	0.437	0.428	48.462***	−0.310	1.815	−0.171	−3.875	3.255
Age				0.002	0.017	0.129	−0.032	0.036
Sex				0.527	0.245	2.151*	0.046	1.009
Living arrangement				0.291	0.345	0.843	−0.387	0.969
Educational attainment				−0.108	0.123	−0.872	−0.350	0.135
Marital status				0.230	0.145	1.590	−0.054	0.514
Economic status				−0.225	0.197	−1.139	−0.613	0.163
Loneliness				0.287	0.031	9.306***	0.226	0.347
Depression				0.255	0.045	5.705***	0.167	0.323
Social participation				−0.011	0.019	−0.616	−0.048	0.025
Dependent variable: QOL								
Constant	0.564	0.556	71.521***	95.962	6.888	13.932***	82.430	109.495
Age				−0.034	0.066	−0.512	−0.164	0.096
Sex				0.687	0.935	0.735	−1.150	2.524
Living arrangement				0.495	1.311	0.378	−2.080	3.070
Educational attainment				−0.383	0.469	−0.817	−1.303	0.538
Marital status				−0.578	0.550	−1.050	−1.660	0.503
Economic status				1.272	0.749	1.698	−0.200	2.744
Loneliness				−0.888	0.127	−7.013***	−1.137	−0.639
Depression				−1.047	0.175	−5.980***	−1.391	−0.703
Anxiety				−0.670	0.170	−3.945***	−1.004	−0.337
Social participation				0.384	0.071	5.451***	0.246	0.523

This table presents the results of the multiple hierarchical regression analysis of social participation, loneliness, depression, anxiety and QOL. Sex, age, living arrangement, Educational attainment, marital status and economic status are the control variables, social participation is an independent variable, and QOL is a result variable. β = unstandardized coefficient. LLCI refers to the lower limit of the 95% confidence interval for the estimated value, and ULCI refers to the upper limit of the 95% confidence interval for the estimated value. *p < 0.05, **p < 0.01, ***p < 0.001.

**Figure 1:**
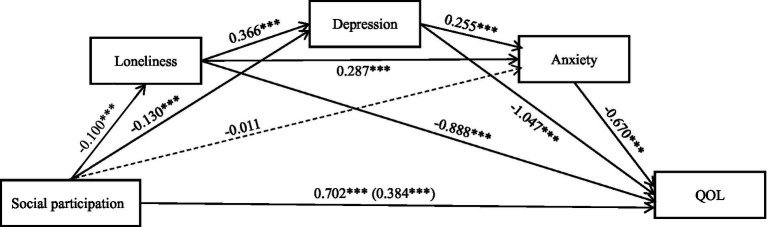
Model regarding the chain mediating effects of loneliness, depression, and anxiety on the relationship between social participation and QOL. The total effect of social participation and QOL is shown in parentheses. All path coefficients are unstandardized. *** *p* < 0.001.

Table 4 shows the indirect effect of loneliness, depression and anxiety between social participation and QOL. Figure 1 presents a chain mediating model of social participation and QOL. Both Table 4 and Figure 1 show that the direct effect of social participation on QOL (*β* = −0.100, *p* < 0.01) is significant, as the 95% confidence interval (CI) [−0.159, −0.041] does not contain zero. Additionally, the total effect of social participation on QOL (*β* = 0.702, *p* < 0.001) is significant, with the 95% CI [0.538, 0.866] excluding zero. Regarding loneliness, the 95% CI [0.015, 0.077] does not contain zero, indicating a significant mediating effect (*β* = 0.089, *p* < 0.001) in the relationship between social participation and QOL, confirming Hypothesis 2. Similarly, depression shows a significant mediating effect (*β* = 0.136, *p* < 0.001), with the 95% CI [0.043, 0.096] excluding zero, confirming Hypothesis 3. However, anxiety does not have a significant mediating effect in this relationship, as evidenced by the 95% CI [−0.007, 0.019] containing zero and a non-significant coefficient (*β* = 0.008, *p* > 0.05). Thus, Hypothesis 4 is rejected.

**Table 4:** Results of the indirect effect based on bootstrapping Test.

Effect	Path	*β*	*SE*	t/z	*p*	LLCI	ULCI	Relative mediation effect
Total indirect effect	Social participation⇒QOL	0.318***	0.033	9.727	0.000	0.100	0.229	82.81%
Indirect effect	Social participation⇒Loneliness⇒QOL	0.089***	0.017	5.357	0.000	0.015	0.077	23.18%
	Social participation⇒Depression⇒QOL	0.136***	0.013	10.255	0.000	0.043	0.096	35.42%
	Social participation⇒Anxiety⇒QOL	0.008	0.006	1.209	0.227	−0.007	0.019	2.08%
	Social participation⇒Loneliness⇒Depression⇒QOL	0.038***	0.007	5.137	0.000	0.007	0.036	9.90%
	Social participation⇒Loneliness⇒Anxiety⇒QOL	0.019***	0.004	4.369	0.000	0.003	0.019	4.95%
	Social participation⇒Depression⇒Anxiety⇒QOL	0.022***	0.004	5.568	0.000	0.004	0.020	5.73%
	Social participation⇒Loneliness⇒Depression⇒Anxiety⇒QOL	0.006***	0.001	4.228	0.000	0.001	0.007	1.56%

β = unstandardized coefficient; LLCI refers to the lower limit of the 95% confidence interval for the estimated value, and ULCI refers to the upper limit of the 95% confidence interval for the estimated value; ***p < 0.001.

Furthermore, the chain mediation effects involving loneliness and depression in the relationship between social participation and QOL are significant, with a 95% CI [0.007, 0.036] excluding zero (*β* = 0.038, *p* < 0.001). The chain mediation effect of loneliness and anxiety is also significant, with a 95% CI [0.003, 0.019] not containing zero (*β* = 0.019, *p* < 0.001). Additionally, depression and anxiety show a significant chain mediation effect, with a 95% CI [0.004, 0.020] excluding zero (*β* = 0.022, *p* < 0.001). Lastly, the chain mediation involving loneliness, depression, and anxiety is significant, as the 95% CI [0.001, 0.007] does not contain zero (*β* = 0.006, *p* < 0.001). Hypotheses 5 is confirmed.

## 4 Discussion

This study empirically developed and validated a research model to investigate the internal mechanisms between social participation and QOL among older adults in China.

First, the study results confirmed a significant positive relationship between social participation and QOL, aligning with prior research in this area (36). Specifically, higher levels of social participation and active involvement in various interpersonal and sociocultural activities were shown to enhance QOL. This suggests that social participation plays a crucial role in promoting the wellbeing of older adults in China.

Second, this study found that social participation can significantly affect QOL through individual mediators such as loneliness and depression. However, when anxiety was tested as an independent mediator in the relationship between social participation and QOL, it did not exhibit a significant mediating effect. Unlike previous research, which often focused on the simple linear relationships between social participation and these psychological factors (19, 32–35), this study explored their distinct roles as independent mediators in the relationship between social participation and QOL. By analyzing loneliness, depression, and anxiety separately, the findings provide a deeper understanding of the underlying mechanisms at play, thus contributing to the existing body of knowledge in this field.

Through the implementation of chain mediation analyses, this study further demonstrated the sequential mediating effects of loneliness, depression, and anxiety in the relationship between social participation and QOL. The results indicate that the QOL of older adults in China is not solely directly influenced by social participation but also indirectly shaped by the cumulative impact of these psychological health factors, which are influenced by social participation. Interestingly, although anxiety did not show a significant mediating effect when tested independently in the relationship between social participation and QOL, it became significant when examined in conjunction with loneliness and depression as part of a chain mediation model. This suggests that while social participation may not directly impact QOL through anxiety, it may lead to increased loneliness, which exacerbates depression levels, subsequently heightening anxiety and ultimately reducing QOL. Thus, when different psychological health indicators are included in the model as internal mechanisms, social participation’s effect on loneliness and depression is more direct. In contrast, the increase in anxiety is more a result of heightened loneliness and depression. Regardless, the adverse psychological health consequences of insufficient social participation significantly affect QOL, highlighting the need for attention and intervention.

The finding of chain mediation analyses can be interpreted from a cultural perspective. China, as a collectivist country, emphasizes group survival, where people are interdependent (44). This contrasts with the individualistic culture of Western countries, which is characterized by greater separation from the group, more distance between individuals within the group, and a focus on self-reliance, individuality, and independence (45). In comparison, older adults in China, embedded in a collectivist culture, are more accustomed to communal living and value connections with others. Therefore, they are more likely to achieve self-fulfillment and satisfaction through various social interactions (46). Upon retirement, they may need to maintain good psychological health through social participation and interpersonal interactions, ensuring satisfactory QOL. This chain mediation model offers deeper insights into the complex mechanisms through which social participation impacts QOL, contributing to a more nuanced understanding of the interplay between these variables in enhancing the wellbeing of older adults in China.

The practical implications of this study are significant. Firstly, enhancing social participation is crucial for helping older adults in China achieve better QOL. Encouraging them to maintain contact and interaction with family and friends and organizing diverse activities at the community level can alter their monotonous lifestyles, encouraging them to engage with the outside world and enhance communication. For instance, activities that allow older Chinese to showcase their skills and talents can help them discover self-worth and boost their confidence. Celebrating major traditional festivals and regional specialties can also foster interactions between community members, corporate employees, and students, creating more connections and mutual understanding with older adults. Over time, these activities can evolve into organized projects, developing volunteer teams and forming a stable social service framework for the older adults.

Attention should also be given to those older adults who, due to disabilities or illnesses, find it challenging to participate in social activities. These individuals often have a monotonous daily routine and limited interaction with the outside world, making them more susceptible to loneliness, depression, and anxiety, which can deteriorate their QOL. Solutions to help these socially isolated older adults could include encouraging them to use popular social media platforms like WeChat and QQ for online social participation (29), thereby improving their health and wellbeing. Providing timely psychological support and emotional counseling services can also prevent a decline in their QOL.

This study has some limitations that need addressing. First, due to difficulties in sample collection, a convenience sampling method was used, and the sample was limited to Guangzhou China, potentially affecting the sample’s representativeness. Future research should expand the sampling scope and optimize sampling methods to enhance the study’s representativeness and generalizability. Second, this study conducted a cross-sectional analysis, focusing on the social participation, psychological health status, and QOL of older adults at a specific stage, and constructed models and mediation analyses based on these data. It did not capture the changes in social participation, psychological health, and QOL over different age stages. Future research could attempt longitudinal analyses to better understand the dynamics of these variables. Furthermore, this study did not categorize older adults to compare their social participation, health conditions, and QOL. Given the significant differences in abilities, economic conditions, and living arrangements among the older population, future research should classify older adults based on these characteristics and conduct comparative studies for a deeper understanding of the research topic.

## Data Availability

The original contributions presented in the study are included in the article/supplementary material, further inquiries can be directed to the corresponding author.
